# Immersive Reality–Based Training Simulator for Dental Extraction: Protocol for a Randomized Pilot Trial

**DOI:** 10.2196/74978

**Published:** 2025-11-05

**Authors:** Endang Sjamsudin, Muhammad Ruslin, Olivia Avriyanti Hanafiah, Carolina Stevanie, Sri Hastuti Kurniawan, Muh Anshar, Paolo Boffano, Tymour Forouzanfar, Cortino Sukotjo

**Affiliations:** 1 Department of Oral and Maxillofacial Surgery Faculty of Dentistry Padjajaran University Bandung Indonesia; 2 Department of Oral and Maxillofacial Surgery Faculty of Dentistry Hasanuddin University Makassar Indonesia; 3 Department of Oral and Maxillofacial Surgery Faculty of Dentistry University of North Sumatra Medan Indonesia; 4 Department of Computational Media Jack Baskin School of Engineering University of California, Santa Cruz Santa Cruz, CA United States; 5 Department of Electrical Engineering Faculty of Engineering Hasanuddin University Makassar Indonesia; 6 Department of Health Sciences Università degli Studi del Piemonte Orientale “Amedeo Avogadro” Novara Italy; 7 Department of Oral and Maxillofacial Surgery Leiden University Medical Center Leiden The Netherlands; 8 Department of Prosthodontics School of Dental Medicine University of Pittsburgh Pittsburgh, PA United States

**Keywords:** dental extraction, immersive reality, pilot trial, virtual training, study protocol

## Abstract

**Background:**

Dental students’ competencies are shaped by their training, yet traditional methods with mannequins often lack the depth necessary for comprehensive understanding, potentially impacting clinical proficiency. Immersive reality (IR) innovatively offers interactive and scenario-based environments that may enhance skill acquisition.

**Objective:**

This study evaluates the effectiveness of IR-based training implementation in comparison with conventional training methods for dental extractions.

**Methods:**

A prospective multicenter randomized clinical trial was conducted. Students were randomized to either IR-based training on open and closed extractions or conventional hands-on tutorials by oral surgeons. Post training, participants’ satisfaction and understanding were assessed and analyzed.

**Results:**

As of September 2025, 60 students from Hasanuddin University, Makassar, and Padjajaran University, Bandung, have been enrolled, and study enrollment will be expanded to Universitas Sumatera Utara, Medan. Data collection is ongoing and will conclude in November 2025, with expected dissemination in early 2026.

**Conclusions:**

IR-based training offers a novel approach that may boost motivation, knowledge retention, and skill transfer in dental education. This pilot protocol explores IR’s feasibility and potential to advance dental students’ competencies.

**Trial Registration:**

Indonesian Clinical Research Registry INA-QES4CC5; https://ina-crr.kemkes.go.id/en/studi/207

**International Registered Report Identifier (IRRID):**

DERR1-10.2196/74978

## Introduction

Dental extraction is among the most frequently performed procedures in dental practice [1]. Indications for extraction are diverse, including periodontitis, endodontic complications, orthodontic needs, ectopic eruption, prosthetic treatment planning, traumatic injury, and systemic conditions, with dental caries being the most common cause [2-4]. The high prevalence of extractions underscores the importance of equipping dental students with sufficient knowledge, technical proficiency, and clinical competence [5,6].

Conventional training methods and passive observation restrict students’ ability to understand the complexity of treatments, particularly within the confined working environment of the oral cavity [7,8]. Moreover, novice dental students often lack familiarity with assisting tasks, reducing training efficiency and learning outcomes. Effective skill acquisition requires not only theoretical knowledge but also spatial imagination, active engagement, and repeated practice opportunities [9-11]. Consequently, extensive training and prolonged clinical exposure are often necessary before dental students achieve the level of competence required to provide safe and effective oral health care [12,13].

Immersive reality (IR), which integrates virtual reality and augmented reality, has emerged as a promising educational technology to overcome these challenges [14-17]. IR provides a safe, realistic, and hands-on platform for students to practice clinical procedures and refine their skills [18-25]. Evidence suggests that IR-based training improves practical competence, treatment planning, and standardized assessment while offering repeatable and engaging learning experiences [26-30]. Importantly, IR technology supports consistent training for complex procedures that demand precision and manual dexterity [31-33]. By addressing limitations of conventional learning—such as high costs, limited patient cases, and restricted opportunities for repeated practice—IR can bridge gaps in skill acquisition, foster collaboration, and enhance creativity in clinical training [34-38].

Despite these advantages, the application of IR as a simulator for dental extraction training remains insufficiently explored. To date, IR technology has been implemented in operative dentistry, periodontology, and dental radiography for practices such as tooth drilling, endodontic cavity access, crown placement, and periodontal procedures [39-43]. Furthermore, none of these applications involves students from multicenter institutions. This study seeks to highlight the use of IR training for dental extractions involving students from multicenter institutions while also generating feedback for further refinement of the simulator and its associated training protocol.

## Methods

### Apparatus

Unity 3D Engine software (Microsoft Corporation) was used as the platform for simulating dental extraction procedures in an IR environment. This platform enables users to engage from a first-person perspective. In the virtual environment, users can interact with 3D models by using either hand gestures or controllers.

In this study, the IR hardware setup consists of a personal computer featuring an Intel Core i7-11800H chipset and an NVIDIA GeForce RTX 3080 graphics card, which includes a GPU with 10 GB of GDDR6X memory. The configuration also incorporates a Meta Quest Pro 3 (Reality Labs) head-mounted display (HMD). The equipment enables Unity to run at a constant frame rate of over 60 frames per second using the “high quality” display settings of the HMD.

### Trial Design and Study Population

#### Overview

This protocol is for a prospective multicenter randomized clinical trial, which began in March 2025 and is expected to end in November 2025. This protocol outlines our plan to assess the knowledge and skill enhancement of a pilot intervention across three centers: Hasanuddin University (Makassar, Indonesia), Padjajaran University (Bandung, Indonesia), and Universitas Sumatera Utara (Medan, Indonesia). A total of 90 dental students participated in the study (G*Power effect size 0.6, statistical power 85%). All participants enrolled in this study were randomly assigned to either an intervention group (n=15) or a control group (n=15) within each center. We used the block randomization method for the allocation of participants.

#### Blinding and Calibration of Reviewer

Two independent reviewers (an oral and maxillofacial surgeon) were assigned to evaluate and score each participant’s pretest and posttest sheets (Multimedia Appendix 1). Before data collection, the assessors underwent a calibration session to standardize the answer key for the multiple-choice questions (MCQs) and ensure consistent scoring. During the study, the assessors were blinded to the participants’ identities and allocation status, as they did not have access to names or group assignments. A final score for each participant was calculated.

Additionally, the reviewer will assess the participants’ training videos, focusing on training time completion, in-app lag or confusion, and skipped steps, to evaluate participants’ performance during training.

#### Informed Consent

Before participating in this study, the research assistant explained the trial flow and procedures for both the IR-based training and the conventional training. All participants will receive a brief introduction to the study and will have the opportunity to ask questions about the study procedures. After the participants provide their written informed consent (Multimedia Appendix 2), their basic health status will be recorded (Multimedia Appendix 3). Participants who are unable to give consent will be excluded from the study.

#### Inclusion Criteria

Participants will be enrolled from the Universitas Sumatera Utara, Hasanuddin University, and Padjajaran University. Eligible participants must be 20 years or older and be currently enrolled in their third year of dental school or have completed a minimum of seven semesters. They should have completed tutorials on both the open and closed methods of tooth extraction, possess knowledge of medical technology, and be capable of providing informed consent. Individuals who are unable to engage with virtual content for a minimum duration of 10 minutes while using an HMD will be excluded from participation.

#### Exclusion Criteria

Individuals who are unable to provide informed consent, possess health complications, display symptoms of cybersickness (eg, dizziness, disorientation, or vomiting) during training sessions, or are unable to complete the training session are excluded from participation. Additional exclusion criteria encompass a history of hallucinations, panic attacks, seizures, phobias (including claustrophobia and acrophobia), or active alcohol or substance abuse.

#### Intervention Group

Before the intervention, two oral and maxillofacial surgeons from each center will follow a comprehensive meeting held in Hasanuddin University, Makassar, to ensure consistent delivery of the interventions across centers and instructors. In addition, a facility assessment will be reported during the meeting to ensure that each center can deliver the intervention method using the same protocol.

Participants will be given a brief introduction and explanation of the study’s purpose. A research assistant will present the informed consent both in writing and verbally. This study has adopted standardized procedures for tooth extraction, using both open and closed methods.

The IR training session will be conducted in a standing position, where participants role-play as dentists. The training session will focus on open and closed dental extraction for the first molar tooth (tooth 46), supervised by two oral and maxillofacial surgeons in each center. For all training sessions, the first 10 minutes will be conducted under direct supervision. Participants will then continue their sessions for the last 40 minutes using in-app assistance (sound, highlighted areas, and arrows) without additional support for the remaining session.

The IR HMD wirelessly streams to an external device and will be used during the first session to monitor the participants’ training execution and provide guidance when necessary. Participants were required to complete a full round of training. This procedure included several steps: administering local anesthesia, making an incision and separating the tooth (in open method extraction mode), luxating and extracting the tooth, performing curettage, irrigating the wound, and suturing.

Following the completion of the procedure, participants are presented with the option to either restart the training sessions by selecting the reset button or terminate the session by choosing the exit button located in the menu area.

#### Control Group

**Figure 1:**
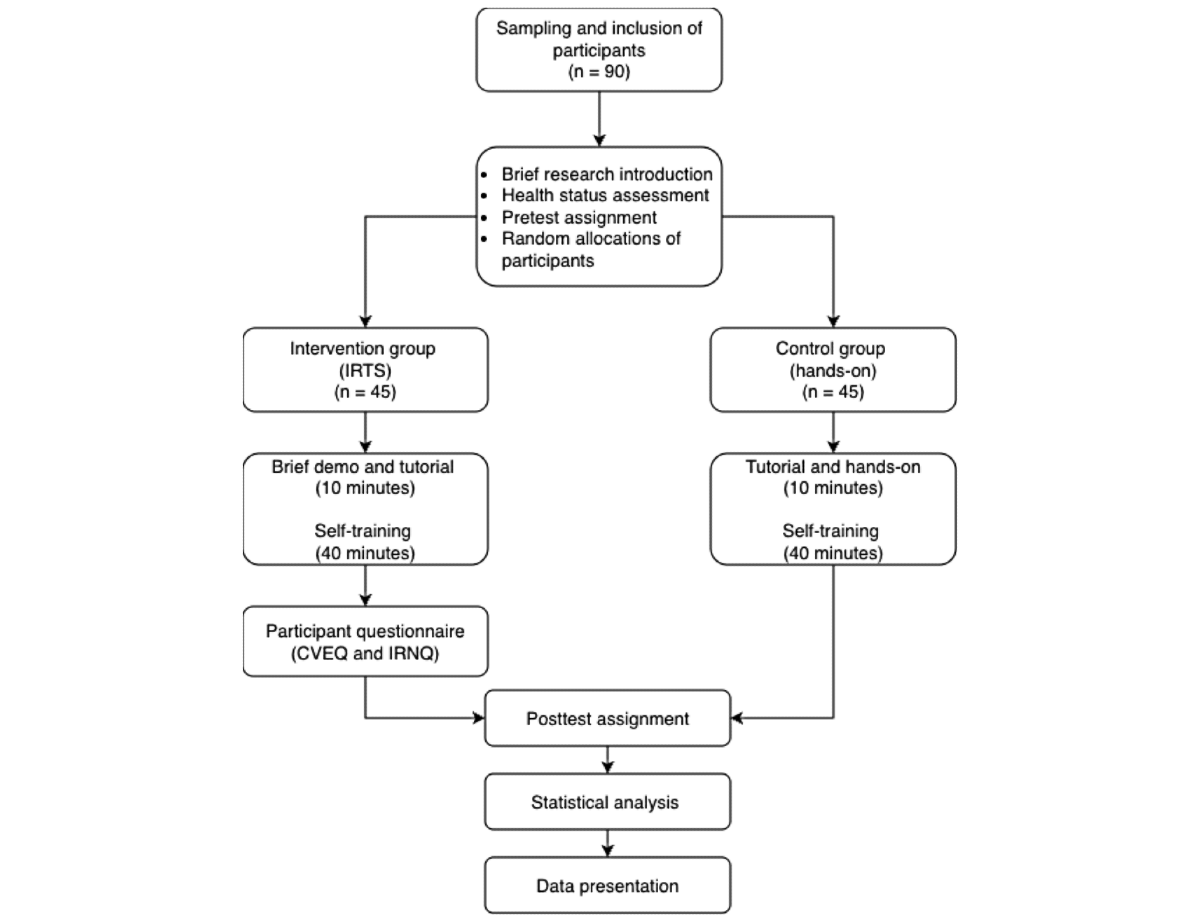
Research flowchart. CVEQ: Clinical Virtual Evaluation Questionnaire; INRQ: Immersive Reality Neuroscience Questionnaire; IRTS: Immersive Reality Training System.

Participants in the control group visited the Department of Oral and Maxillofacial Surgery at their origin center (Hasanuddin University, Padjajaran University, or Universitas Sumatera Utara) to receive a tutorial and hands-on laboratory instructions. Following a 10-minute lecture on the open and closed methods of dental extraction for the first molar tooth (tooth 46), participants will engage in hands-on training where they will extract artificial teeth from a phantom or mannequin, much like in the IR-based instructions. The hands-on training will have a duration of 40 minutes, starting from local anesthesia administration, gingival incision, tooth separation, tooth luxation, extraction, curettage, wound irrigation, and suturing. Tutorial and hands-on training will be guided by two oral and maxillofacial surgeons in each center. The trial flowchart is shown in Figure 1. To ensure a standardized methodology for conducting and reporting the trial, the SPIRIT (Standard Protocol Items for Intervention Trials) guidelines will be followed as shown in Table 1.

**Table 1:** SPIRIT (Standard Protocol Items for Intervention Trials) participant timeline.

Timepoint		Enrollment (t_–1_ to t_0_)	t_0_	Postrandomization (t_1_; May 2025)	Closeout (t_x_; Nov 2025)
**Enrollment**					
	Eligibility screen	t_–1_			
	Informed consent	t_–1_			
	Baseline assessment (MCQs^a^)	✓			
	Randomization	✓			
**Intervention/comparator**					
	IRTS^b^ for tooth extraction		Tutorial of tooth extraction	✓	✓
	Hands-on training		Tutorial of tooth extraction	✓	✓
**Assessments**					
	MCQ questionnaires	✓			✓
	Knowledge improvement, posttest scores				✓
	Participants’ satisfaction with IRTS				✓

^a^MCQ: multiple-choice question.

^b^IRTS: Immersive Reality Training System.

#### Adverse Events

Participants who report feeling nauseated, dizzy, or disoriented, or having other health problems during a training session, will immediately be excluded from the activity. All such complaints will be recorded as cybersickness, and the occurrence of the condition will be noted for future evaluation.

### Data Collection

#### Participants’ Experience and Feedback

This study used two distinct questionnaires to assess the participants’ perspectives toward the usability of the Immersive Reality Training System (IRTS) for dental extraction training, which were designed based on previous research [44]. The Clinical Virtual Evaluation Questionnaire is structured to gather participants’ feedback on interactive education facilitated through simulator training (Multimedia Appendix 4). This questionnaire assesses three key parameters: experience and reliability (questions 1-9), knowledge (questions 10-14), and side effects (question 15). The questionnaire comprises 15 statements, which respondents assess using a Likert scale ranging from 1 (strongly disagree) to 5 (strongly agree). Conversely, the Immersive Reality Neuroscience Questionnaire will assess the system’s authenticity, the participants’ experiences, and the occurrence of cybersickness (Multimedia Appendix 5). This assessment will concentrate on four principal parameters: user experience (questions 1-5), game mechanism (questions 6-10), in-app assistance (questions 11-15), and IR-induced symptoms and effects (questions 16-20). The questionnaire is composed of 20 questions, which participants will answer using a Likert scale ranging from 1 (extremely poor) to 7 (extremely good). To ensure that the system’s quality is deemed suitable for use without causing significant cybersickness, each parameter must meet a minimum threshold (cutoff) score of 25 [45].

#### Participants’ Performance and Knowledge Improvement

To assess the practical skills and knowledge acquired, participants from both the intervention and control groups will be required to complete a pretest prior to the training and a posttest following the training session. The MCQs were administered in accordance with the reference textbook used by the Faculty of Dentistry, as outlined in the dentistry curriculum [46]. The scores ranged from 0 to 100, and the test scores of both groups were subsequently compared. The MCQs are centered on the procedural steps, instrumentation, and guiding principles pertinent to dental extraction. This examination is an established component of the curricula at Hasanuddin University, Padjajaran University, and Universitas Sumatera Utara, aimed at assessing students’ practical competencies and theoretical knowledge. Consequently, a comparative analysis test will be used to evaluate the scores obtained from the clinical skill training between these two groups.

Additionally, the performance of participants will be evaluated through recorded video training and will be assessed by an independent reviewer, focusing on training time completion, in-app lag or confusion, and skipped steps. The confidential paper-based data of participants will be securely stored in a locked storage unit at the study site, while electronic data will be maintained in a protected folder on a server at Hasanuddin University, Makassar, Indonesia.

### Data Analysis Plan

The data were analyzed using SPSS v.22 (IBM Corp). Both descriptive statistics (frequency, percentage, mean, and SD) and inferential statistics (multivariate ANOVA) were used. A *P* value less than .05 was considered statistically significant.

### Ethical Considerations

This study was approved by the Ethics Committee of the Faculty of Dentistry at Hasanuddin University in Makassar, Indonesia, in October 2024 (approval 001/KEPK FKG-RSGMP UH/EE/IX/2024). Written consent was obtained from all participants, and they were also assured that their information would be kept completely confidential. This manuscript is in accordance with the SPIRIT 2025 guidelines (Multimedia Appendix 6) [47].

## Results

Study enrollment started in May 2025 and is expected to be completed in November 2025. As of October 2025, we have enrolled 60 participants from two centers (Hasanuddin University, Makassar, and Padjajaran University, Bandung) and will continue recruiting from a third center (Universitas Sumatera Utara, Medan).

The trial was registered in the Indonesian Clinical Research Registry (INA-QES4CC5) on November 18, 2024.

## Discussion

### Strengths and Limitations

This study uses IR technology to advance the training of dental students in dental extraction methods, allowing them to experience immersive, high-fidelity scenarios that closely resemble actual clinical situations. One of the primary strengths of the IR training program is its incorporation of essential procedures for both open and closed extraction. The adaptation and validation process involves a thorough selection procedure conducted in collaboration with engineering experts. Consequently, the findings will offer both quantitative and qualitative perspectives on the enhancement of participants’ abilities. Moreover, the research included dental students from three distinct provinces (West Sumatra, West Java, and South Sulawesi) to provide an in-depth analysis of the potential influence of IR-based training on dental students’ skills in performing tooth extractions.

The presence of a control group in this study enables the observation of improvements in dental students. The pre- and posttest MCQs allow not only comparisons within the same group but also evaluation of the IRTS against traditional methods regarding dental students’ learning outcomes, thereby strengthening the validity of our findings.

In recognizing the strengths of this study, it is equally crucial to address its limitations. A notable concern is the potential for selection bias, as participants may inherently possess a favorable disposition toward technology or virtual reality environments. To mitigate this bias, we have meticulously documented the participants’ prior interactions with virtual reality technology and incorporated this information into experience distribution tables.

### Challenges

One of the primary challenges in this study is the adaptation of the IR environment to align with actual dental extraction procedures. The IR environment differs from conventional simulation in its representation of communication and interaction, as certain elements, such as natural communication with patients and their relatives, may be absent, while others, including nonverbal cues and nuanced interpersonal behaviors, are often not effectively captured within the IR environment. To address this concern, the IRTS has been developed, featuring interactive questions aimed at enhancing students’ comprehension of patient diagnoses. Moreover, evaluators face the ongoing challenge of accurately observing and assessing participant behavior in an IR scenario. To consistently and objectively implement the IRTS, it is essential to have a clear and thorough understanding of participant behaviors, both as they occur in real time and through recorded observations. One significant challenge associated with the implementation of IR technologies in dental education is the cost of procurement [48]. However, recent publications indicate that while the initial investment in IR technology may be substantial, it offers the advantage of providing repeatable training without the need for physical materials. Consequently, it can be used in the long term to reduce the costs associated with practical models for tutorials and hands-on training [49]. As the technology becomes more affordable and user-friendly, its applicability in medical and dental training is expected to grow [49].

### Future Implications

In addressing certain limitations of the study, future research could investigate the incorporation of more advanced haptic devices and natural language processing to enhance the realism of the IR environment and more comprehensively capture the full spectrum of participant behavior. As IR technology continues to progress, it is imperative to consistently refine the tools used to ensure they remain congruent with the evolving capabilities of the technology.

## Data Availability

The datasets generated or analyzed during this study will be made publicly available after completion of the study and will be available from the corresponding author upon reasonable request.

## Appendix

Multimedia Appendix 1 Pretest and posttest multiple-choice questions.

Multimedia Appendix 2 Informed consent form.

Multimedia Appendix 3 Health status form.

Multimedia Appendix 4 Clinical Virtual Evaluation Questionnaire.

Multimedia Appendix 5 Immersive Reality Neuroscience Questionnaire.

Multimedia Appendix 6 SPIRIT 2025 checklist.

## Abbreviations

HMD: head-mounted display
IR: immersive reality
IRTS: Immersive Reality Training System
MCQ: multiple-choice question
SPIRIT: Standard Protocol Items for Intervention Trials
