# Supplementary material for: Immersive Reality–Based Training Simulator for Dental Extraction: Protocol for a Randomized Pilot Trial
Source: JMIR Res Protoc. 2025 Nov 5;14:e74978. doi: 10.2196/74978 (PMC12631091; doi:10.2196/74978)
Supplement: Multimedia Appendix 1 [file resprot_v14i1e74978_app1.docx]

**PRE-TEST**

**Dental Extraction Procedures**

| **Date of assignment:** |
| --- |
| 1. The first procedural step in closed extraction of an erupted tooth is: A. Forceps adaptation B. Luxation with elevator C. Soft tissue reflection/loosening D. Application of traction forces  2. The main purpose of reflecting the gingival papilla before elevator placement is to: A. Reduce bleeding B. Avoid damaging adjacent tissue C. Increase alveolar bone expansion D. Prevent tooth fracture  3. During luxation with a straight elevator, excessive buccal elevation is avoided because it may cause: A. Loss of clot stability B. Buccal bone fracture C. Root resorption D. Premature tooth rotation  4. The proper initial site for elevator placement is: A. Buccal bone surface B. Mesial and distal interdental spaces C. Apical root tip D. Lingual plate  5. The beaks of extraction forceps should be adapted: A. To the crown above the gingival margin B. To the root surface, apical to the cervical line C. To the occlusal third of the crown D. To the interdental papilla  6. The correct sequence of force application with forceps is: A. Traction → Apical pressure → Buccolingual expansion B. Apical pressure → Buccolingual expansion → Rotational/traction forces C. Buccolingual expansion → Rotational → Apical pressure D. Apical pressure → Traction only  7. Apical pressure with forceps primarily serves to: A. Expand alveolar bone B. Luxate the tooth immediately C. Seat beaks onto root surface D. Prevent crown fracture  8. Rotational forces are indicated mainly for: A. Multirooted molars B. Single-rooted conical teeth (e.g., maxillary central incisor) C. Mandibular first molars D. All maxillary premolars  9. The final force used to deliver a tooth from the socket is: A. Apical pressure B. Buccolingual expansion C. Traction (outward pull) D. Rotational force  10. When extracting maxillary molars, the typical path of delivery is: A. Straight occlusal B. Buccal and occlusal direction C. Lingual direction D. Rotational only  11. Sectioning of teeth is usually indicated when: A. Roots are widely divergent B. Crown is intact and strong C. Single conical root exists D. Patient has thin alveolar bone  12. In open extraction, after flap reflection, bone removal should typically extend: A. One-third to two-thirds of root length B. Only to cervical level C. To full root apex D. Minimal coronal third only  13. Entry points are created in surgical extraction to: A. Place sutures B. Seat forceps beaks more apically C. Engage elevator tips for controlled removal D. Reduce crown resistance  14. When sectioning a mandibular molar, the crown is usually divided: A. Mesiodistally into buccal and lingual halves B. Into mesial and distal root portions C. At the cervical line D. Into three equal root sections  15. A common elevator used in sectioned root removal is: A. Coupland elevator B. Cryer elevator C. Warwick-James elevator D. Cowhorn forceps  16. The purpose of applying constant, slow force instead of sudden jerks is to: A. Minimize pain perception B. Reduce risk of alveolar fracture C. Increase periodontal ligament tearing D. Reduce haemorrhage  17. When multiple adjacent teeth are to be extracted, the recommended sequence is: A. Posterior to anterior B. Anterior to posterior C. Random to distribute forces D. Opposite arches alternately  18. After extraction, initial haemostasis is achieved by: A. Suctioning socket blood B. Irrigation with saline C. Placement of gauze pack with biting pressure D. Suture closure always  19. A critical inspection step after tooth removal is to: A. Immediately irrigate socket with hydrogen peroxide B. Check socket for remaining root tips, bone fragments, or debris C. Pack socket with antibiotic ointment D. Probe alveolus with sharp instrument  20. Sutures after surgical extraction should be tied: A. Very tightly to prevent bleeding B. Loosely to approximate tissue without ischemia C. Across the occlusal table for stability D. Over intact bone only |
| **Score*:** |

*a correct answer to each question earns 5 points

**POST-TEST**

**Dental Extraction Procedures**

| **Date of assignment:** |
| --- |
| 1. The first procedural step in closed extraction of an erupted tooth is: A. Forceps adaptation B. Luxation with elevator C. Soft tissue reflection/loosening D. Application of traction forces  2. The main purpose of reflecting the gingival papilla before elevator placement is to: A. Reduce bleeding B. Avoid damaging adjacent tissue C. Increase alveolar bone expansion D. Prevent tooth fracture  3. During luxation with a straight elevator, excessive buccal elevation is avoided because it may cause: A. Loss of clot stability B. Buccal bone fracture C. Root resorption D. Premature tooth rotation  4. The proper initial site for elevator placement is: A. Buccal bone surface B. Mesial and distal interdental spaces C. Apical root tip D. Lingual plate  5. The beaks of extraction forceps should be adapted: A. To the crown above the gingival margin B. To the root surface, apical to the cervical line C. To the occlusal third of the crown D. To the interdental papilla  6. The correct sequence of force application with forceps is: A. Traction → Apical pressure → Buccolingual expansion B. Apical pressure → Buccolingual expansion → Rotational/traction forces C. Buccolingual expansion → Rotational → Apical pressure D. Apical pressure → Traction only  7. Apical pressure with forceps primarily serves to: A. Expand alveolar bone B. Luxate the tooth immediately C. Seat beaks onto root surface D. Prevent crown fracture  8. Rotational forces are indicated mainly for: A. Multirooted molars B. Single-rooted conical teeth (e.g., maxillary central incisor) C. Mandibular first molars D. All maxillary premolars  9. The final force used to deliver a tooth from the socket is: A. Apical pressure B. Buccolingual expansion C. Traction (outward pull) D. Rotational force  10. When extracting maxillary molars, the typical path of delivery is: A. Straight occlusal B. Buccal and occlusal direction C. Lingual direction D. Rotational only  11. Sectioning of teeth is usually indicated when: A. Roots are widely divergent B. Crown is intact and strong C. Single conical root exists D. Patient has thin alveolar bone  12. In open extraction, after flap reflection, bone removal should typically extend: A. One-third to two-thirds of root length B. Only to cervical level C. To full root apex D. Minimal coronal third only  13. Entry points are created in surgical extraction to: A. Place sutures B. Seat forceps beaks more apically C. Engage elevator tips for controlled removal D. Reduce crown resistance  14. When sectioning a mandibular molar, the crown is usually divided: A. Mesiodistally into buccal and lingual halves B. Into mesial and distal root portions C. At the cervical line D. Into three equal root sections **Answer: B**  15. A common elevator used in sectioned root removal is: A. Coupland elevator B. Cryer elevator C. Warwick-James elevator D. Cowhorn forceps  16. The purpose of applying constant, slow force instead of sudden jerks is to: A. Minimize pain perception B. Reduce risk of alveolar fracture C. Increase periodontal ligament tearing D. Reduce haemorrhage  17. When multiple adjacent teeth are to be extracted, the recommended sequence is: A. Posterior to anterior B. Anterior to posterior C. Random to distribute forces D. Opposite arches alternately  18. After extraction, initial haemostasis is achieved by: A. Suctioning socket blood B. Irrigation with saline C. Placement of gauze pack with biting pressure D. Suture closure always  19. A critical inspection step after tooth removal is to: A. Immediately irrigate socket with hydrogen peroxide B. Check socket for remaining root tips, bone fragments, or debris C. Pack socket with antibiotic ointment D. Probe alveolus with sharp instrument  20. Sutures after surgical extraction should be tied: A. Very tightly to prevent bleeding B. Loosely to approximate tissue without ischemia C. Across the occlusal table for stability D. Over intact bone only |
| **Score*:** |

*a correct answer to each question earns 5 points

**Key Answer**

1.C 2.B 3.B 4.B 5.B 6.B 7.C 8.B 9.C 10.B

11.A 12.A 13.C 14.B 15.B 16.B 17.A 18.C 19.B 20.B

**Source:** Hupp JR, Ellis E, Tucker MR. Chapter 8: Principles of Routine Exodontia in Contemporary Oral and Maxillofacial Surgery. 2019. 7^th^ ed. Elsevier.
