# Supplementary material for: Immersive Reality–Based Training Simulator for Dental Extraction: Protocol for a Randomized Pilot Trial
Source: JMIR Res Protoc. 2025 Nov 5;14:e74978. doi: 10.2196/74978 (PMC12631091; doi:10.2196/74978)
Supplement: Multimedia Appendix 2 [file resprot_v14i1e74978_app2.pdf]

INFORMED CONSENT FORM

CLIENT INFORMATION

Full Name:

Date of Birth: Gender:

Address:

City: State/Province:

Zip/Postal Code: Country:

Phone Number: Email Address:

GENERAL STATEMENT OF PROGRAM OBJECTIVES AND PROCEDURES:

- This training program aims to improve understanding and skills related to dental extraction procedure using immersive reality technology. This procedure included several steps: Administering local anesthesia, making an incision and separating the tooth (in open method extraction mode), luxating and extracting the tooth, performing curettage, irrigating the wound and suturing. Afterward, participants were asked to complete questionnaires to assess their experience and outcomes after the training.

DESCRIPTION OF POTENTIAL RISKS:

- The usage of head-mounted displays may cause dizziness, disorientation, and headaches in some people.
  For your convenience, participants who wear glasses may continue to wear them.

CONSENT STATEMENT:

- After hearing/reading and understanding the explanation given regarding the purpose, benefits, and what will be done in this study, I agree to participate in this research voluntarily without coercion. I know that my participation is voluntary without coercion, so I can refuse to participate or withdraw from this study. I have the right to ask questions or ask for clarification from researchers if there are things that are unclear or if there are things I want to know about this research.
- I also understand that the security and confidentiality of research data will be guaranteed and I hereby consent to all of my data produced in this research to be presented in verbal or written form.

By affixing my signature below, I confirm my voluntary participation in this research study.

Participant's Signature:

Participant's name:
