# Supplementary material for: Immersive Reality–Based Training Simulator for Dental Extraction: Protocol for a Randomized Pilot Trial
Source: JMIR Res Protoc. 2025 Nov 5;14:e74978. doi: 10.2196/74978 (PMC12631091; doi:10.2196/74978)
Supplement: Multimedia Appendix 3 [file resprot_v14i1e74978_app3.pdf]

HEALTH STATUS FORM

Date of Consultation: ____________

Patient Information

Full Name: ____________

Phone Number: ____________

Date of Birth: ____________

Email: ____________

Gender:

☐ Male ☐ Female ☐ Other

Emergency Contact Name & Phone: ____________

Medical Examination (For Doctor Use)

Blood Pressure: ____________ mmHg

Temperature: ____________

Heart Rate: ____________ bpm

Weight: ____________ [illegible]

History of systemic disease

Do you claim to have a systemic disease? ☐ Yes ☐ No

Type of disease ____________

On treatment ☐ Yes ☐ No

Drugs consume routinely:

History of phobia or neurologic syndrome

Do you claim to have phobia or mental disorders? ☐ Yes ☐ No

Type of phobia or mental disorder ____________

On treatment ☐ Yes ☐ No

Drugs consume routinely:

Doctor's statement

Elligible to be sample ☐ Yes ☐ No

Note:

Doctor's Signature: ____________

Doctor's Name:
