# Supplementary material for: Immersive Reality–Based Training Simulator for Dental Extraction: Protocol for a Randomized Pilot Trial
Source: JMIR Res Protoc. 2025 Nov 5;14:e74978. doi: 10.2196/74978 (PMC12631091; doi:10.2196/74978)
Supplement: Multimedia Appendix 4 [file resprot_v14i1e74978_app4.pdf]

CLINICAL-VIRTUAL EVALUATION QUESTIONNAIRE (CVEQ)

PERSONAL INFORMATION:

NAME: ____________________

DATE OF BIRTH: DAY / MONTH / YEAR ____________________

EDUCATION LEVEL ____________________

GENDER: ○ Male ○ Female ____________________ Not Listed ○ Experienced in immersive reality technology

ADDRESS:

Unit No.	St. No.	Street	Town/City/Suburb	State	Zip Code	Country

INSTRUCTIONS:

Choose one of the following rating scale that express your experience about immersive reality system

QUESTIONS:	Strongly disagree	Disagree	Neutral	Agree	Strongly agree
I like this method of learning and training.	○	○	○	○	○
I quickly and easily understood the orthognathic surgery procedure.	○	○	○	○	○
The surgery is displayed accurately and appropriately in the system.	○	○	○	○	○
The operating room environment is accurately and appropriately displayed in the system.	○	○	○	○	○
Instruments are displayed accurately and appropriately in the system.	○	○	○	○	○
I was able to complete the operator role in surgery very well.	○	○	○	○	○
The guidelines and steps were not difficult for me at all.	○	○	○	○	○
I am satisfied with the teaching methods and training I received.	○	○	○	○	○
This app can be used as an initial practical knowledge of orthognathic surgery.	○	○	○	○	○
Based on the training content and the understanding I obtained, I can clearly understand the anatomical structure of the orthognathic surgical area.	○	○	○	○	○
This teaching method can effectively help me understand orthognathic surgical procedures and deepen my understanding of orthognathic surgery.	○	○	○	○	○
This training really helped me learn the stages of orthognathic surgical procedures.	○	○	○	○	○
This training was very helpful for me in learning the difficulties in orthognathic surgical procedures.	○	○	○	○	○
Based on the training content and the understanding I gained, I believe I can independently perform orthognathic surgical design in the future.	○	○	○	○	○
I felt dizzy during/after the training	○	○	○	○	○
