# Supplementary material for: Immersive Reality–Based Training Simulator for Dental Extraction: Protocol for a Randomized Pilot Trial
Source: JMIR Res Protoc. 2025 Nov 5;14:e74978. doi: 10.2196/74978 (PMC12631091; doi:10.2196/74978)
Supplement: Multimedia Appendix 5 [file resprot_v14i1e74978_app5.pdf]

IMMERSIVE REALITY NEUROSCIENCE QUESTIONNAIRE (IRNQ)

PERSONAL INFORMATION:

NAME: ______________________

DATE OF BIRTH: DAY / MONTH / YEAR

EDUCATION LEVEL ______________________

GENDER: ◯ Male ◯ Female ______________________

◯ Experienced in immersive reality technology

ADDRESS:

Unit No.	St. No.	Street	Town/City/Suburb	State	Zip Code	Country

INSTRUCTIONS:

Choose one of the following rating scale that express your experience about immersive reality system

QUESTIONS:	Extremely poor	Very Poor	Poor	Enough	Good	Very good	Extremely good
How do you experience the level of immersivity	◯	◯	◯	◯	◯	◯	◯
How satisfied are you with your immersive reality (IR) experience	◯	◯	◯	◯	◯	◯	◯
How is the graphic quality	◯	◯	◯	◯	◯	◯	◯
How is the sound quality	◯	◯	◯	◯	◯	◯	◯
How is the overall quality of immersive reality (IR) technology (hardware and other supporting devices)	◯	◯	◯	◯	◯	◯	◯
How easy is it to use the navigation system in the immersive reality environment	◯	◯	◯	◯	◯	◯	◯
How easy it is to be physically move around in the immersive reality environment	◯	◯	◯	◯	◯	◯	◯
How easy it is to pick up and/or place items in an immersive reality environment.	◯	◯	◯	◯	◯	◯	◯
How easy it is to use items in the immersive reality environment	◯	◯	◯	◯	◯	◯	◯
How easy is it to perform two-handed interactions, such as holding a model with one hand and another object with the other in an immersive reality environment	◯	◯	◯	◯	◯	◯	◯
How easy is it to complete the orthognathic surgery tutorial in the app	◯	◯	◯	◯	◯	◯	◯
How useful was the tutorial provided	◯	◯	◯	◯	◯	◯	◯
What do you think about the tutorial duration	◯	◯	◯	◯	◯	◯	◯
How helpful were the in-app instructions for completing the tutorial	◯	◯	◯	◯	◯	◯	◯
How easy to understand are the in-app instructions	◯	◯	◯	◯	◯	◯	◯
Do you feel any nausea	◯	◯	◯	◯	◯	◯	◯
Do you feel any disorientation	◯	◯	◯	◯	◯	◯	◯
Do you feel any fatigue	◯	◯	◯	◯	◯	◯	◯
Do you feel any instability	◯	◯	◯	◯	◯	◯	◯
